# Longer survival and favorable healthcare resource utilization of tagraxofusp versus venetoclax in patients with blastic plasmacytoid dendritic cell neoplasm: results of a real-world analysis

**DOI:** 10.3389/fonc.2026.1903818

**Published:** 2026-08-12

**Authors:** Anthony S. Stein, Guru Subramanian Guru Murthy, Pankit Vachhani, Sunil Iyer, Deborah Goldschmidt, Yipeng Gao, John Katsetos, Corey Pelletier, Tibor Kovacsovics

**Affiliations:** 1 City of Hope, Duarte, CA, United States; 2 Medical College of Wisconsin, Milwaukee, WI, United States; 3 University of Alabama at Birmingham, Birmingham, AL, United States; 4 Columbia University, New York, NY, United States; 5 Analysis Group, Inc., New York, NY, United States; 6 Analysis Group, Inc., Boston, MA, United States; 7 Menarini Group, New York, NY, United States; 8 City of Hope, Goodyear, AZ, United States

**Keywords:** BPDCN, healthcare resource utilization, real-world, survival, tagraxofusp, venetoclax

## Abstract

**Background:**

Tagraxofusp is a first-in-class CD123-targeted therapy approved for blastic plasmacytoid dendritic cell neoplasm (BPDCN). Although venetoclax is not approved for BPDCN, limited use has been reported. We evaluated overall survival (OS) and healthcare resource utilization in patients with BPDCN treated with tagraxofusp- or venetoclax-based regimens in a US real-world setting.

**Methods:**

Claims data were analyzed from patients with ≥1 BPDCN diagnosis (January 2016-December 2023) and initiation of tagraxofusp- or venetoclax-based therapy. Index date was defined as first inpatient tagraxofusp dosing or venetoclax-based therapy initiation. Patients were matched 1:1 by line of therapy (LOT).

**Results:**

Each cohort had 47 patients. Tagraxofusp-based treatment was associated with longer median OS versus venetoclax-based (35.3 vs 10.5 months), consistent across patients receiving monotherapy, combination, or transplantation. Tagraxofusp-treated patients were more likely to proceed to subsequent LOT versus venetoclax-treated (47% vs 11%). During months 2-6, tagraxofusp reduced median inpatient hospitalization duration (3 vs 7 days) and transfusion rates (3% vs 32%) versus venetoclax.

**Conclusion:**

In this retrospective claims-based analysis, tagraxofusp was associated with longer survival versus venetoclax, including across treatment contexts. These real-world results are consistent with the established role of tagraxofusp as the first-line standard of care for patients with BPDCN, irrespective of transplant eligibility, and support continued evaluation of tagraxofusp-based regimens as a backbone for future combinations.

## 1 Introduction

Patients with blastic plasmacytoid dendritic cell neoplasm (BPDCN), an aggressive, orphan hematologic malignancy characterized by cells expressing CD123 and other markers, frequently present with skin, bone marrow, blood, and visceral involvement, and face a poor prognosis (1–4). The goal of first-line treatment for these patients is to use systemic therapy to induce rapid and durable complete remission (CR) without long-term toxicities, especially myelosuppression (5, 6). Achievement of such remission enables timely transition for eligible patients to hematopoietic stem cell transplantation (HSCT), which remains the only potentially curative option. For those who are ineligible for HSCT, the goal is to continue maintenance therapy to prolong remission without cumulative toxicity. Until the 2018 approval of the first-in-class CD123-targeted agent tagraxofusp, the first approved treatment for BPDCN, patients were treated with intensive chemotherapy regimens not approved for BPDCN and developed for other hematologic malignancies (4, 7). Moreover, the studies of intensive chemotherapy were retrospective and lacked prespecified multisystem response criteria and measurement tools. Responses to these chemotherapy regimens have lacked durability, and many patients with BPDCN are unable to receive intensive induction chemotherapy due to age or fitness. The outlook for all patients with BPDCN, including unfit and older patients, has improved since tagraxofusp became the first drug approved for adults or children age 2 years or older with treatment-naïve or relapsed/refractory BPDCN in the United States (8), and for adults with treatment-naïve BPDCN in Europe (9).

The approval of tagraxofusp was based on a multicenter phase 2 trial (NCT02113982), the largest prospective BPDCN study to date, which used predefined multisystem response criteria (10, 11). In this trial, the 65 patients treated with first-line tagraxofusp (12 µg/kg once daily on days 1–5 of a 21-day cycle) had a 75% overall response rate (ORR) and a 57% CR/clinical CR (CRc; CR with residual skin abnormalities not indicative of active BPDCN) rate, with a median overall survival (OS) of 15.8 months (95% confidence interval [CI], 9.7-25.8) (11). Furthermore, 51% of patients with CR/CRc and two additional patients with partial response transitioned to HSCT, with a median OS not reached (NR; range, 3.5-58.1 months) at a median follow-up since transplant of 34 months (12). Complete and durable responses have also been seen irrespective of a patient’s age or pre-treatment fitness level (13, 14). Similarly, in a real-world study, 26 treatment-naïve patients with BPDCN treated with first-line tagraxofusp had a 90% ORR and 65% CR rate, with a median OS of 20.2 months (95% CI, 10.2-not estimable [NE]) (15). The 50% of patients who bridged to HSCT had a median OS of 37.0 months (95% CI, 10.7-NE) (15).

Tagraxofusp has a well-characterized and manageable safety profile. Treatment with tagraxofusp is marked by transient adverse events that occur mostly in cycle 1, with no cumulative long-term toxicity, no myelosuppression, and rapid restoration of hematopoiesis during treatment with tagraxofusp in the majority of patients irrespective of baseline bone marrow involvement (11, 15, 16). Capillary leak syndrome (CLS) has been observed with tagraxofusp. In the pivotal study, 21% of patients reported CLS, which was mostly grade 2 (67%) (11). Real-world and clinical trial experience demonstrate that with careful monitoring for early signs and appropriate intervention during tagraxofusp treatment, CLS is usually manageable and in most cases mild, limited to the first cycle, and does not recur (11, 15, 17). The unique bone marrow–sparing effects of tagraxofusp have been substantiated by a decreased requirement for both platelet and red blood cell transfusions from baseline over the course of tagraxofusp therapy in the pivotal trial (16). The triplet combination of tagraxofusp with venetoclax and azacitidine has been studied for both treatment-naïve and relapsed/refractory BPDCN in a phase 2 trial (18). In treatment-naïve patients, both median progression-free survival and OS were NR, and the safety profile of the triplet was tolerable and consistent with previous observations with tagraxofusp and venetoclax-azacitidine.

Venetoclax, an oral BCL2 inhibitor, given in combination with a hypomethylating agent (HMA) is approved as a first-line regimen for patients with acute myeloid leukemia (AML) who are not eligible to receive intensive chemotherapy (19). The use of venetoclax in AML along with research showing BCL2 dependence in BPDCN cells spurred its off-label clinical use to treat BPDCN (20). Clinical data in first-line and relapsed/refractory populations have been limited, with results published in case reports (21–27), retrospective cohort studies (28, 29), a retrospective case series (30), and, most recently, a report of ten patients with relapsed/refractory BPDCN prospectively treated in two clinical trials (31).

There are no prospective studies comparing regulatory-approved tagraxofusp and off-label venetoclax treatment in patients with BPDCN. In a retrospective cohort study using a global health records database, Lee found similar 1-year survival outcomes with venetoclax plus HMA compared with tagraxofusp monotherapy in older patients with BPDCN (30). Whereas the two cohorts of patients shared some baseline demographic and medical history characteristics, data on treatment history and detailed comorbidities that could inform treatment planning were lacking (30). To provide more data to help guide treatment decisions, we performed a retrospective, observational cohort study evaluating OS and healthcare resource utilization (HCRU) in patients with BPDCN matched by prior line of therapy (LOT) and treated with tagraxofusp- or venetoclax-based regimens in US real-world settings.

## 2 Methods

### 2.1 Data source

The Komodo Research Database (KRD) was used to analyze deidentified medical and pharmacy claims data. The KRD is a large, US-based administrative claims database comprising data from over 150 payers and multiple claims processors, capturing approximately 330 million unique patient journeys. It is broadly representative of the US population across age, income, race, ethnicity, and insurance types. The database includes information on diagnoses, procedures, treatments, provider and payer characteristics, mortality, and demographic variables. As a deidentified database, the KRD adheres to article 164.514 of the US Health Insurance Portability and Accountability Act. As such, institutional review board exemption and patient consent were not required for this study.

### 2.2 Study population

**Figure 1:**
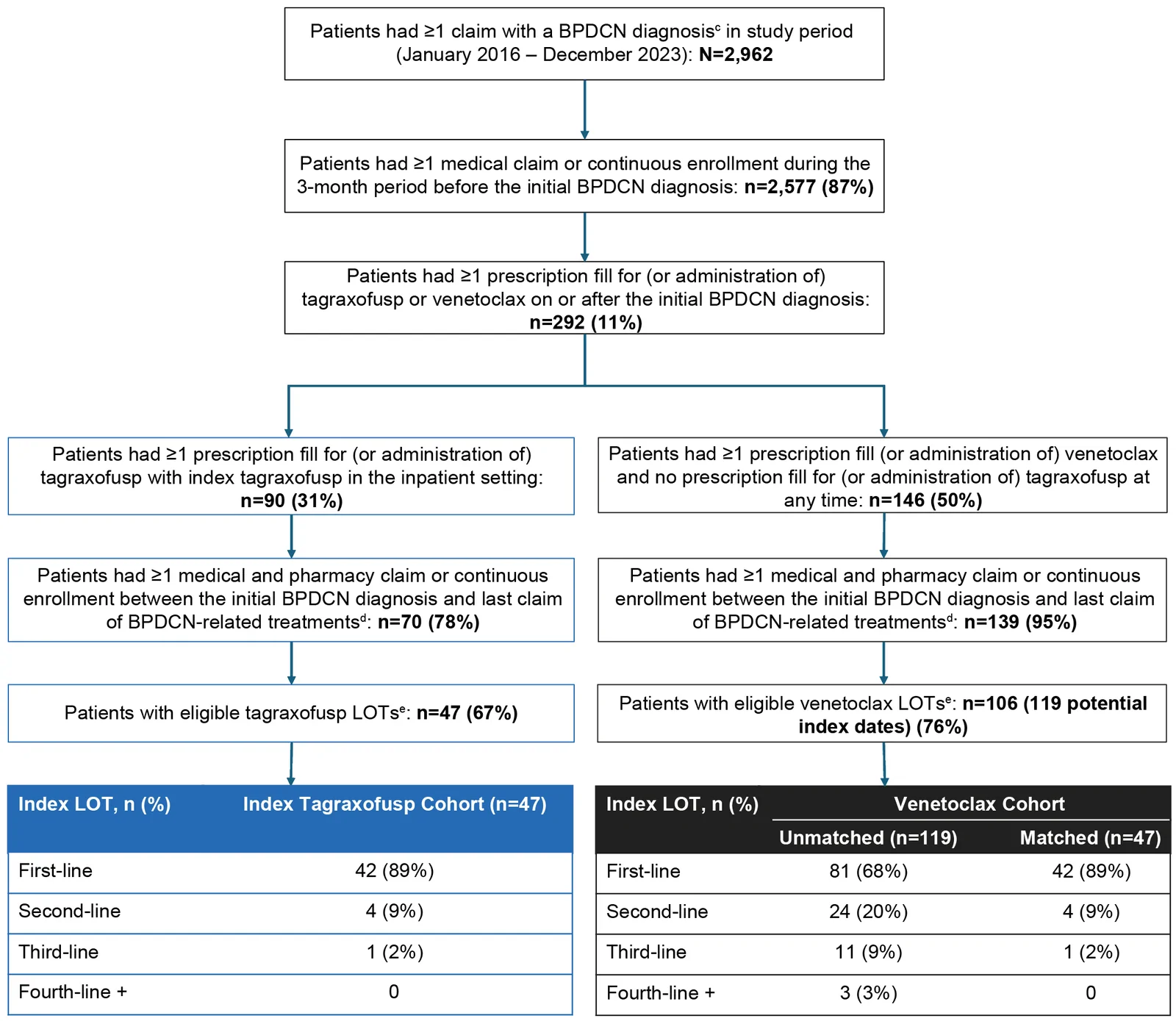
Selection of study population. ^a,b^ Flow diagram of the selection process used to form the tagraxofusp- and venetoclax-based study cohorts. BPDCN, blastic plasmacytoid dendritic cell neoplasm; ICD-10-CM, International Classification of Diseases, Tenth Revision, Clinical Modification; LOT, line of therapy. ^a^Pharmaceutical claims were restricted to paid claims only. ^b^Medical and pharmacy claims were restricted to full-view data from January 1, 2016, to December 4, 2023. ^c^ICD-10-CM BPDCN diagnosis code C86.4. ^d^BPDCN-related treatments included tagraxofusp-based therapies, venetoclax-based therapies, and BPDCN-directed chemotherapies. ^e^Eligible LOTs were defined as (1) ≥1 claim for tagraxofusp or venetoclax with non-missing days of supply; and (2) initiation of the LOT with tagraxofusp or venetoclax ≥30 days before the later of the end of continuous medical and pharmacy eligibility or the last observed claim for BPDCN-related treatment.

Our analysis included records of patients with at least one International Classification of Diseases, Tenth Revision, Clinical Modification (ICD-10-CM) diagnosis code for BPDCN (C86.4) between January 2016 and December 2023, subsequent initiation of a tagraxofusp-based regimen in an inpatient setting or of a venetoclax-based regimen in any setting (**Figure 1**). The index date was defined as the date of first inpatient administration of a tagraxofusp or initiation of a venetoclax-based regimen. To ensure complete capture of BPDCN-related treatment history, eligible patients were also required to have continuous pharmacy and medical enrollment or evidence of medical claims activity from 3 months prior to the initial BPDCN diagnosis through the last observed claim for a BPDCN-related treatment. Patients were also required to have an eligible tagraxofusp or venetoclax LOT, defined as those meeting the following criteria: (1) at least one claim for tagraxofusp or venetoclax with non-missing days of supply; and (2) initiation of the LOT at least 30 days before the later of the end of continuous medical and pharmacy eligibility or the last observed claim for BPDCN-related treatment. Patients who died within 30 days of LOT initiation were retained in the analysis. From the eligible population, two cohorts of patients were identified. One cohort comprised patients who initiated a tagraxofusp-based regimen in an inpatient setting, and one cohort comprised patients who received a venetoclax-based regimen and no evidence of tagraxofusp use at any time. To ensure comparability between cohorts in terms of disease severity and treatment history, patients in the tagraxofusp-based cohort were matched 1:1 with patients in the venetoclax-based cohort on the number of prior LOTs, determined using a claims-based algorithm.

### 2.3 Outcomes and analysis

Demographic characteristics were assessed on the index date. Comorbidities were assessed during the 6-month pre-index period using the Charlson Comorbidity Index (CCI) (32, 33). Prior or concomitant hematologic malignancies (PCHM) were identified during the 3-month period prior to the initial BPDCN diagnosis. Follow-up was defined as the time from the index date to the end of data availability (i.e., the later of the end of continuous enrollment or the date of the first claim with a ≥90-day claim-free gap [for prescription claims, a ≥90-day claim-free gap after the last day of supply]), or death, whichever occurred first.

Overall survival was measured from the index date to death from any cause and analyzed separately for the tagraxofusp- and venetoclax-based cohorts in the overall population and in the following subgroups: stratification by post-index HSCT, regimen type (monotherapy ± intrathecal chemotherapy vs combination therapy), patients receiving index treatment in the first-line setting, patients receiving intrathecal chemotherapy (as prophylaxis or treatment unspecified), and patients in the venetoclax-based cohort who received HMAs within the index LOT.

All-cause HCRU metrics included inpatient stays, inpatient days and length of stay (LOS), emergency room (ER) visits, outpatient visits, and receipt of blood product transfusions. These metrics were evaluated from the index date to the earliest of end of index LOT, end of data availability, or death, up to 6 months. Healthcare resource utilization was summarized monthly among patients with any follow-up for that month. Additionally, HCRU during months 2–6 post-index was summarized on a per-patient-per-month (PPPM) and per-patient-per-hospitalization (PPPH) basis to account for varying follow-up time. In addition, inpatient days and LOS were calculated among patients who had at least one inpatient stay during the specified time period. Primary diagnoses associated with ER visits during months 2–6 post-index were also summarized by cohort.

### 2.4 Statistical methods

This was a retrospective, descriptive, cohort study. Continuous variables were summarized using means and standard deviations (SDs) or medians and interquartile ranges (IQRs); categorical variables were described as counts and percentages. Kaplan-Meier methods were used to estimate OS, with median OS and the corresponding two-sided 95% CI derived using the Brookmeyer and Crowley method (34). Patients without a death event were censored at the last date of data availability. A sensitivity analysis censored patients at the earlier of either the last date of data availability or the date of first post-index HSCT. Healthcare resource utilization outcomes were summarized descriptively as monthly rates and on a PPPM and PPPH basis for months 2-6, separately by cohort. All statistical analyses were conducted using SAS software, version 9.4.

## 3 Results

### 3.1 Patient demographics and baseline characteristics

Forty-seven patients treated with tagraxofusp-based regimens and 47 patients treated with venetoclax-based regimens were included in this analysis (**Table 1**). The mean patient age was 60.9 years for the tagraxofusp-based cohort and 67.3 years for the venetoclax-based cohort. Mean CCI scores were 0.8 for the tagraxofusp-based cohort and 1.1 for the venetoclax-based cohort. Common CCI comorbidities for patients receiving tagraxofusp-based regimens and venetoclax-based regimens, respectively, included diabetes (30% vs 40%), peripheral vascular disease (13% vs 26%), and chronic pulmonary disease (21% vs 15%).

**Table 1:** Characteristics of patients in the line of therapy–matched tagraxofusp-based and venetoclax-based cohorts.

Characteristic	Tagraxofusp-based cohort (N = 47)	Venetoclax-based cohort (N = 47)
Age (years),^a^		
Mean ± SD	60.9 ± 19.6	67.3 ± 18.8
Sex,^a^ n (%)		
Female	13 (28)	8 (17)
Male	34 (72)	39 (83)
Race/ethnicity,^a^ n (%)		
White or Caucasian	26 (55)	30 (64)
Black or African American	6 (13)	3 (6)
Asian or Pacific Islander	0 (0)	3 (6)
Hispanic or Latino	7 (15)	3 (6)
Other	2 (4)	3 (6)
Unknown	6 (13)	5 (11)
Year of index date, n (%)		
2017	–	0 (0)
2018	–	9 (19)
2019	5 (11)	7 (15)
2020	13 (28)	13 (28)
2021	7 (15)	6 (13)
2022	11 (23)	1 (2)
2023	11 (23)	10 (21)
BPDCN duration at index (months),^b^		
Mean ± SD	4.2 ± 8.1	5.9 ± 9.0
Prior or concomitant additional hematologic malignancies,^c^ n (%)		
Multiple myeloma	2 (4)	6 (13)
Myelodysplastic syndromes	4 (9)	4 (9)
Acute lymphoblastic leukemia	3 (6)	8 (17)
Chronic myelomonocytic leukemia	2 (4)	1 (2)
Hodgkin lymphoma	0 (0)	1 (2)
CCI comorbidity score (excluding cancer),^d^		
Mean ± SD	0.8 ± 1.3	1.1 ± 1.5
CCI comorbidities in >5% of patients,^d^ n (%)		
Diabetes without chronic complication	10 (21)	14 (30)
Chronic pulmonary disease	10 (21)	7 (15)
Peripheral vascular disease	6 (13)	12 (26)
Mild liver disease	5 (11)	6 (13)
Congestive heart failure	5 (11)	9 (19)
Renal disease	5 (11)	4 (9)
Diabetes with chronic complication	4 (9)	5 (11)
Cerebrovascular disease	3 (6)	7 (15)
Myocardial infarction	2 (4)	3 (6)

BPDCN, blastic plasmacytoid dendritic cell neoplasm; CCI, Charlson Comorbidity Index; SD, standard deviation.

^a^ Measured at index date.

^b^ BPDCN duration at index was defined as time from the initial BPDCN diagnosis to the index date.

^c^ Measured during the 90-day period prior to the initial BPDCN diagnosis.

^d^ Measured during the 6-month baseline period prior to the index date.

### 3.2 Treatment patterns

Monotherapy was used in 85% of patients in the tagraxofusp-based cohort and 49% in the venetoclax-based cohort (**Table 2**). Of 24 patients who received a venetoclax-based combination regimen, 21 received venetoclax with HMAs. Intrathecal chemotherapy was administered in 34% of patients in the tagraxofusp-based cohort and 19% in the venetoclax-based cohort. Twelve (26%) patients in the tagraxofusp-based cohort and 13 (28%) in the venetoclax-based cohort proceeded to HSCT.

**Table 2:** Treatment patterns of patients in the line of therapy–matched tagraxofusp-based and venetoclax-based cohorts^a^.

Characteristic	Tagraxofusp-based cohort (N = 47)	Venetoclax-based cohort (N = 47)
Index LOT regimen, n (%)		
Monotherapy^b^	40 (85)	23 (49)
Combination therapy	7 (15)	24 (51)
Venetoclax + HMA	–	21 (45)
Received IT chemotherapy during index LOT, n (%)	16 (34)	9 (19)
IT methotrexate	15 (32)	7 (15)
IT cytarabine	8 (17)	8 (17)
Treatment regimens received as index LOT among all tagraxofusp patients, n (%)		
Tagraxofusp monotherapy	28 (60)	–
Tagraxofusp combination^c^	3 (6)	–
Tagraxofusp monotherapy + IT chemotherapy^d^	12 (26)	–
Tagraxofusp combination + IT chemotherapy^e^	4 (9)	–
Treatment regimens received as index LOT among all venetoclax patients, n (%)		
Venetoclax monotherapy	–	22 (47)
Venetoclax combination	–	16 (34)
Venetoclax monotherapy + IT chemotherapy^g^	–	1 (2)
Venetoclax combination + IT chemotherapy^h^	–	8 (17)
Proceeded to any HSCT, n (%)	12 (26)	13 (28)
Allogeneic only	12 (26)	12 (26
Autologous only	0	1 (2)

BPDCN, blastic plasmacytoid dendritic cell neoplasm; HMA, hypomethylating agent; HSCT, hematopoietic stem cell transplantation; IT, intrathecal; LOT, lines of therapy.

^a^ Treatment patterns and LOTs were identified from the initial BPDCN diagnosis until the later of (a) the end of continuous eligibility or (b) the date of the last claim for BPDCN-related treatment.

^b^ Monotherapy was defined as treatment with tagraxofusp or venetoclax alone, or in combination with IT chemotherapy.

^c^ Tagraxofusp + azacitidine (n = 1), tagraxofusp + venetoclax (n = 1), tagraxofusp + cytarabine (n = 1).

^d^ Tagraxofusp + IT methotrexate (n = 6), tagraxofusp + IT cytarabine + IT methotrexate (n = 5), tagraxofusp + IT cytarabine (n=1).

^e^ Tagraxofusp + venetoclax + IT cytarabine + IT methotrexate (n = 2), tagraxofusp + daunorubicin + vincristine + IT methotrexate (n = 1), tagraxofusp + venetoclax + IT methotrexate (n = 1).

^f^ Venetoclax + azacitidine (n = 9), venetoclax + decitabine (n = 5), venetoclax + decitabine + fludarabine (n = 1), venetoclax + vincristine (n = 1).

^g^ Venetoclax + IT cytarabine + IT methotrexate (n = 1).

^h^ Venetoclax + azacitidine + IT cytarabine + IT methotrexate (n = 3), venetoclax + azacitidine + IT cytarabine (n = 2), venetoclax + cyclophosphamide + cytarabine + doxorubicin + vincristine + IT cytarabine + IT methotrexate (n = 1), venetoclax + decitabine + IT cytarabine + IT methotrexate (n = 1), venetoclax + vincristine + IT methotrexate (n = 1).

More patients received a subsequent LOT in the tagraxofusp-based cohort than in the venetoclax-based cohort (47% vs 11%). Of 22 patients in the tagraxofusp-based cohort who received a subsequent LOT, 5 patients received a tagraxofusp-based regimen as a subsequent LOT, and 11 patients received a venetoclax-based regimen as a subsequent LOT. Median (IQR) follow-up was 13.4 (5.9, 21.0) months for the tagraxofusp-based cohort and 7.4 (3.5, 11.8) months for the venetoclax-based cohort.

### 3.3 Survival

**Figure 2:**
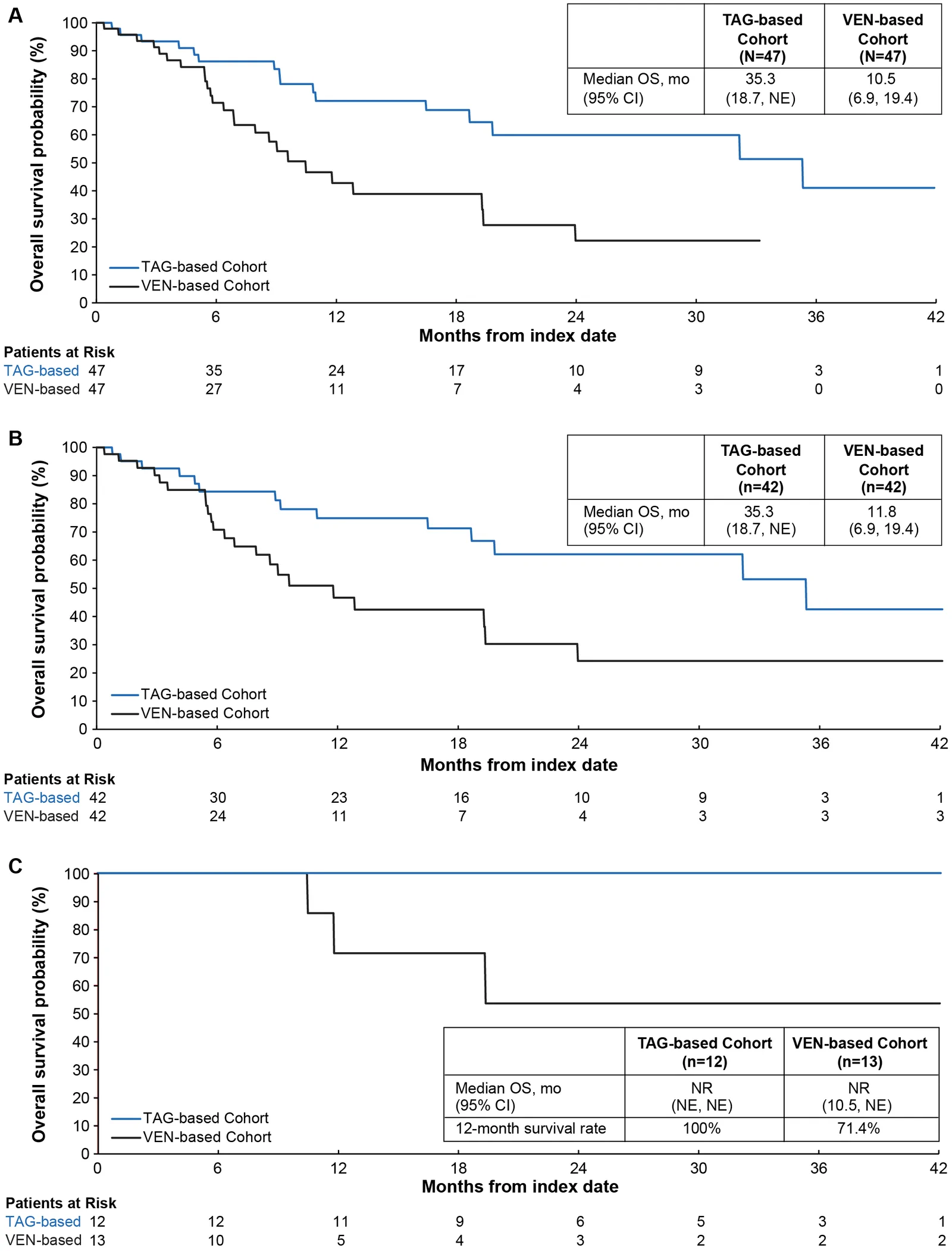
Overall survival by cohort. Kaplan-Meier OS curves of patients treated with tagraxofusp- or venetoclax-based regimens. **(A)** LOT-matched cohorts. **(B)** LOT-matched cohorts in patients receiving the index treatment in the first-line. **(C)** LOT-matched cohorts in patients who proceeded to HSCT. CI, confidence interval; HSCT, hematopoietic stem cell transplantation; LOT, lines of therapy; mo, month; NE, not estimable; NR, not reached; OS, overall survival; TAG, tagraxofusp; VEN, venetoclax.

Median OS was 35.3 months (95% CI, 18.7-NE) for the tagraxofusp-based cohort and 10.5 months (95% CI, 6.9-19.4) for the venetoclax-based cohort (**Figure 2A**), with 12-month survival rates of 72.1% and 42.8%, respectively. In the subgroup of patients receiving the index treatment in the first line, median OS remained longer for the tagraxofusp-based cohort (35.3 months; 95% CI, 18.7-NE) compared with the venetoclax-based cohort (11.8 months; 95% CI, 6.9-19.4) (**Figure 2B**), with 12-month survival rates of 74.9% and 46.7%, respectively. For patients receiving monotherapy, the median OS for the tagraxofusp-based cohort was 35.3 months (95% CI, 18.7-NE) and 10.5 months (95% CI 6.4-19.4) for the venetoclax-based cohort. Median OS in patients treated with combination therapy was NR (95% CI 0.8-NE) for the tagraxofusp-based cohort and 9.6 months (95% CI 5.6-NE) for the venetoclax-based cohort. The median OS in the 21 patients treated with venetoclax plus HMA was 19.3 months (95% CI 5.8-NE).

In the subgroup of patients who proceeded to HSCT, median OS was NR in either cohort (**Figure 2C**), with 12-month survival rates of 100% for tagraxofusp and 71.4% for venetoclax. In the subgroup of patients who did not receive HSCT, median OS was 18.7 months (95% CI, 9.2-NE) for the tagraxofusp-based cohort and 6.9 months (95% CI, 5.6-12.9) for the venetoclax-based cohort, with 12-month survival rates of 59.6% and 33.1%, respectively.

In patients treated with intrathecal chemotherapy, median OS was NR (95% CI, 18.7-NE) in the tagraxofusp-based cohort (n = 16) and NR (95% CI, 2.9-NE) in the venetoclax-based cohort (n=9). Among patients who did not receive intrathecal chemotherapy, median OS was 19.8 months (95% CI, 9.2-NE) for the tagraxofusp-based cohort (n = 31) and 9.6 months (95% CI, 6.4-19.3) for the venetoclax-based cohort (n = 38).

### 3.4 Healthcare resource utilization

Healthcare resource utilization data were available for all 47 patients in each cohort at 1 month post-index and for 33 patients in the tagraxofusp-based cohort and 41 patients in the venetoclax-based cohort at 2–6 months post-index.

**Figure 3:**
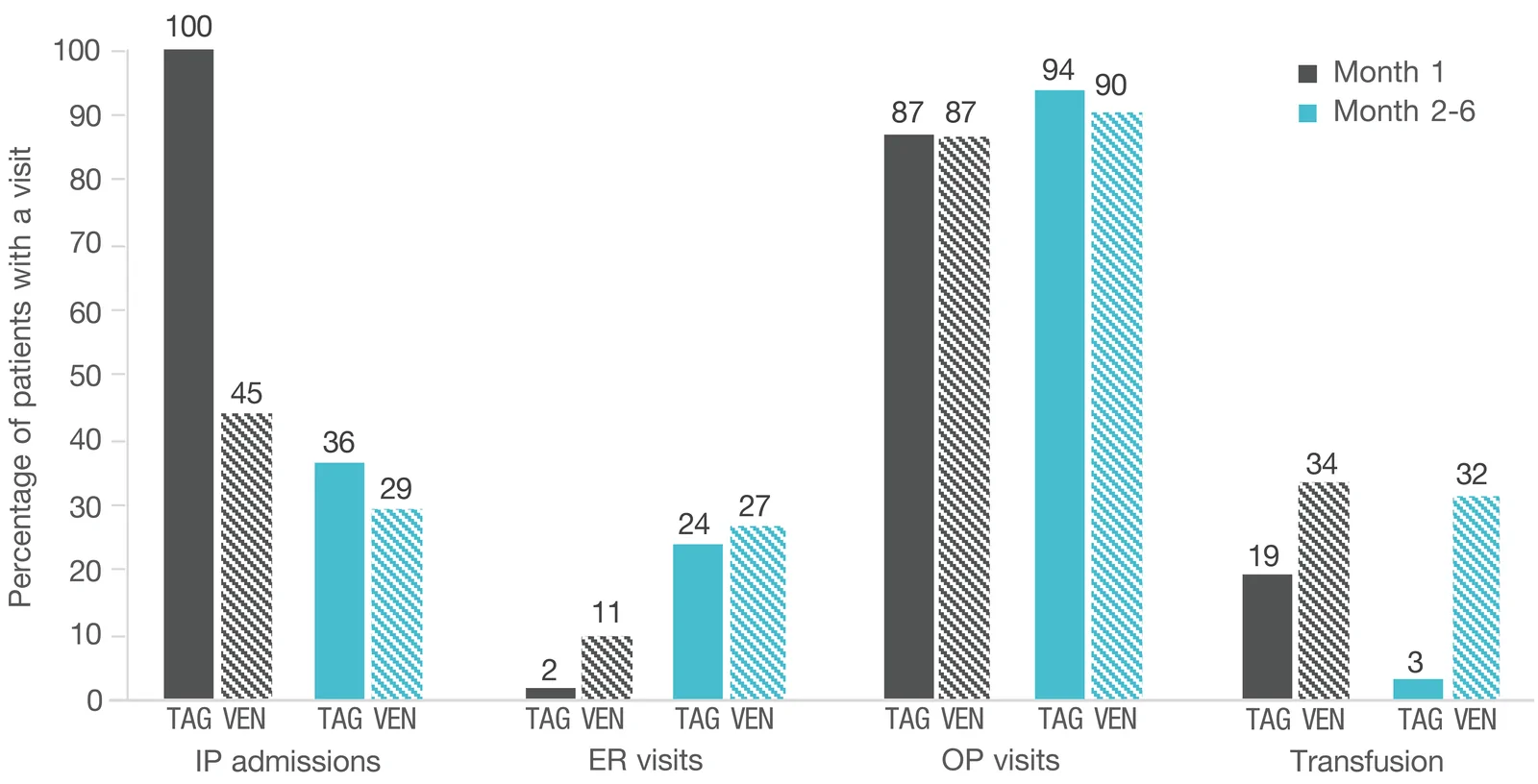
All-cause healthcare resource utilization during follow-up by cohort. All-cause inpatient admissions, ER visits, outpatient visits, and transfusions reported in ≥1 patient in 1 month and 2–6 months after tagraxofusp- or venetoclax-based treatment initiation (index date). ER, emergency room; IP, inpatient; OP, outpatient; TAG, tagraxofusp; VEN, venetoclax.

At 1 month post-index, the tagraxofusp-based cohort had a higher percentage of patients with inpatient admissions (100% vs 45%), reflecting the requirement for hospitalization during tagraxofusp administration in cycle 1, and a lower proportion of patients with ER visits (2% vs 11%) or transfusions (19% vs 34%) compared with the venetoclax-based cohort (**Figure 3**). In both cohorts, 87% of patients had at least one outpatient visit. At 1 month post-index, the two cohorts had a similar median inpatient hospitalization LOS (PPPH; 8.0 vs 6.5 days) and the same 10.8 median total inpatient days PPPM (**Table 3**).

**Table 3:** Healthcare resource utilization by cohort during month 1 and months 2–6 of index line of therapy.

All-cause HCRU, median (IQR)	Month 1 post-index^a^		Months 2–6 post-index^b^	
	Tagraxofusp-based cohort (N = 47)	Venetoclax-based cohort (N = 47)	Tagraxofusp-based cohort (n = 33)	Venetoclax-based cohort (n = 41)
Follow up				
Length of follow-up, months	1 (1.0, 1.0)	1 (1.0, 1.0)	1.6 (1.0, 3.4)	2.1 (1.0, 5.1)
Inpatient admissions				
No. of inpatient admissions, PPPM	1.0 (1.0, 2.0)	0 (0, 1.0)	0 (0, 0.5)	0 (0, 0.1)
LOS, days, PPPH^c^	8.0 (6.0, 13.0)	6.5 (4.0, 11.0)	3.0 (2.0, 7.5)	7.0 (4.3, 24.0)
Total inpatient days, PPPM^c^	10.8 (6.9, 17.7)	10.8 (3.9, 13.7)	3.0 (1.7, 4.8)	5.8 (2.1, 9.0)
No. of ER visits, PPPM				
PPPM	0 (0, 0)	0 (0, 0)	0 (0, 0)	0 (0, 0)
No. of outpatient visits, PPPM^d^				
PPPM^d^	6.9 (2.0, 8.8)	8.8 (2.9, 11.8)	8.8 (4.0, 11.0)	6.6 (2.8, 11.0)
No. of outpatient (other) visits, PPPM^d^				
PPPM^d^	3.9 (1.0, 7.9)	5.9 (2.0, 9.8)	4.0 (1.8, 5.1)	5.8 (2.2, 8.2)
No. of outpatient (BPDCN treatment administration) visits, PPPM^d^				
PPPM^d^	0 (0, 2.0)	0 (0, 3.9)	4.5 (1.0, 6.0)	0.6 (0, 3.4)

BPDCN, blastic plasmacytoid dendritic cell neoplasm; ER, emergency room; HCRU, healthcare resource utilization; IQR, interquartile range; LOS, length of stay; PPPH, per-patient-per-hospitalization; PPPM, per-patient-per-month.

^a^ Measured from the index date to end of data availability, death, or 1 month post-index, whichever was earlier.

^b^ Measured during the follow-up period from month 2 to month 6, defined as the time from 1 month post-index to the earliest of end of continuous eligibility, database availability, death, or 6 months.

^c^ Length of stay and total inpatient days were only calculated among patients with inpatient visits.

^d^ Outpatient visits were categorized as outpatient visits for BPDCN treatment administration (i.e., for tagraxofusp or chemotherapy) and outpatient visits for other reasons. If a patient had both claims for treatment administration and other outpatient visits on the same day, treatment administration was prioritized as the visit type.

At 2–6 months post-index, the tagraxofusp- and venetoclax-based cohorts had similar proportions of patients with inpatient admissions (36% vs 29%), ER visits (24% vs 27%), and outpatient visits (94% vs 90%) (**Figure 3**). Only 3% of patients in the tagraxofusp-based cohort had transfusions compared with 32% in the venetoclax-based cohort. The tagraxofusp-based cohort had a lower median inpatient hospitalization LOS PPPH (3.0 vs 7.0 days), lower median total inpatient days PPPM (3.0 vs 5.8 days), and a similar median number of outpatient visits PPPM (8.8 vs 6.6) compared with the venetoclax-based cohort (**Table 3**).

The most common primary diagnosis of ER visits was hypertension (33%) for the tagraxofusp-based cohort and neutropenia (38%) and other nonspecific abnormal finding of lung field (25%) for the venetoclax-based cohort (**Table 4**).

**Table 4:** Primary diagnosis of emergency room visits at months 2–6 of index line of therapy.

Characteristic	Tagraxofusp-based cohort (n = 33)	Venetoclax-based cohort (n = 41)
No. of patients with any ER visits		
n (%)	8 (24.2)	11 (26.8)
No. of ER visits		
n	12	16
Top primary diagnosis of ER visits (≥2 visits), n (%)^a,b^		
Essential (primary) hypertension	4 (33)	–
Unspecified jaundice	2 (17)	–
Gross hematuria	2 (17)	–
Neutropenia	–	6 (38)
Other nonspecific abnormal finding of lung field	–	4 (25)
Thrombocytopenia, unspecified	–	3 (19)
Sepsis, unspecified organism	–	2 (13)
Other specified types of T/NK-cell lymphoma	–	2 (13)
Acute myeloblastic leukemia, not having achieved remission	–	2 (13)
Myeloid sarcoma, not having achieved remission	–	2 (13)
Atherosclerotic heart disease of native coronary artery without angina pectoris	1 (8)	2 (13)
Pneumonia, unspecified organism	–	2 (13)
Other chest pain	–	2 (13)
Chest pain, unspecified	–	2 (13)

ER, emergency room; NK, natural killer.

^a^ The primary diagnoses listed in this table were not mutually exclusive; a single ER visit might have included multiple diagnoses on the same claim.

^b^ The list is not exhaustive; only primary diagnoses that appeared in ≥2 visits in either the tagraxofusp- or venetoclax-based cohort were included.

## 4 Discussion

In this real-world data cohort study of LOT-matched patients with BPDCN, treatment with tagraxofusp-based regimens was associated with longer median OS (35.3 vs 10.5 months) compared with venetoclax-based regimens, independent of subsequent receipt of HSCT. The survival advantage with tagraxofusp-based therapy was consistent across treatment contexts, including monotherapy, combination regimens, and in patients who underwent HSCT. Notably, a higher proportion of patients treated with tagraxofusp-based regimens proceeded to a subsequent LOT compared with patients treated with venetoclax-based regimens (47% vs 11%, respectively). In the HCRU analysis, a discordance in inpatient hospitalization for tagraxofusp- versus venetoclax-based therapy (100% vs 45%) was noted in month 1 due to the need to monitor for CLS during the tagraxofusp dosing period in cycle 1. During months 2-6, treatment with tagraxofusp-based regimens was associated with both reduced median inpatient hospitalization duration (3 vs 7 days) and lower median total inpatient days (3.0 vs 5.8 days) as well as substantially reduced transfusion rates (3% vs 32%) compared with venetoclax-based regimens, indicating rapid hematopoietic recovery and absence of cumulative myelotoxicity. During this same period, the tagraxofusp- and venetoclax-based cohorts had similar inpatient and ER admission rates. Of particular clinical interest, patients who received a venetoclax-HMA combination (with or without intrathecal chemotherapy) showed inferior outcomes relative to patients who received a tagraxofusp-based regimen. Although patients were not matched by baseline bone marrow function, these results reflect the lack of cumulative myelosuppression and the bone marrow–sparing effects of tagraxofusp observed in the pivotal trial (16). Moreover, these findings support the use of tagraxofusp as the preferred first-line standard of care for patients with BPDCN, irrespective of transplant eligibility.

Although cross-study comparisons must be interpreted with caution, results of our study contrast those reported by Lee in a retrospective database analysis that compared outcomes of venetoclax plus HMA versus tagraxofusp in older patients (mean age, 75 years) with BPDCN (30). Unlike our study that focused on a representative US population and used LOT-matched comparison cohorts, Lee used a global database and did not control for LOT. With a median follow-up of 7.4 months for the venetoclax-based cohort and 9.3 months for the tagraxofusp-based cohort, Lee also reported 12-month survival rates of 41.2% and 53%, respectively (30). In our study, with a similar length of follow-up and a slightly younger population, the 12-month survival rate was 72.1% for the tagraxofusp-based cohort and 42.8% for the venetoclax-based cohort. Notably, Lee did not report on the proportion of patients who proceeded to allogeneic HSCT (30).

These analyses have inherent limitations common to many real-world analyses and applicable to both cohorts. The small sample sizes, though a meaningful cohort in the context of an orphan disease, precluded formal statistical comparisons and limited the ability to adjust for baseline differences, potentially leading to residual confounding, which could have potentially contributed to the survival disparity we observed. Also, OS might have been overestimated because of delayed or incomplete death reporting in databases, though arm bias leading to overestimation of the treatment effect would be unlikely. Due to incomplete records, the reported real-world OS for some patients may be longer than what they experienced. Patients in the venetoclax cohort were older, had a higher baseline CCI score, and also had a shorter follow-up time. Venetoclax-treated patients also had a higher rate of PCHM and were less likely to receive intrathecal prophylaxis. Notably, these claims data do not capture all clinically relevant characteristics, such as performance status, frailty, transplant eligibility, number of sites with disease involvement including the central nervous system, baseline lab values, response to therapy, reasons for treatment selection, or treating center effects. Claims-based analyses are retrospective in nature and have potential patient misclassifications due to billing inaccuracies, coding errors, and use of proxy variables to identify clinical characteristics and treatments. Finally, patient activity may be underestimated because of the use of open claims data, which were needed to achieve a suitable sample size.

## 5 Conclusions

In conclusion, these real-world findings are consistent with the established role of tagraxofusp as first-line standard of care in BPDCN, irrespective of transplant eligibility, and support continued evaluation of tagraxofusp-based regimens. This study also supports the rationale for a tagraxofusp-based backbone for future combination regimens, given its monotherapy activity and lack of cumulative toxicity or myelotoxicity. Ongoing clinical studies are investigating combinations with tagraxofusp to further improve outcomes, including with venetoclax plus azacitidine (NCT03113643) and venetoclax plus multiagent chemotherapy (NCT04216524).

## Data Availability

The data that support the results of this study may be requested for products and the relevant indications that have been authorized by the regulatory authorities in Europe/the United States (or, if not, 2 years have elapsed since the study completion). The Menarini Group will review requests individually to determine whether (1) the requests are legitimate and relevant and meet sound scientific research principles, (2) the requests are within the scope of the participants’ informed consent, and (3) the request is compliant with any applicable law and regulation and with any contractual relationship that Menarini Group and its affiliates and partners have in place with respect to the study and/or the relevant product. Prior to making data available, requestors will be required to agree in writing to certain obligations, including without limitation, compliance with applicable privacy and other laws and regulations. Proposals should be directed to medicalinformation@menarinistemline.com.
